# Lead-it-EAZY! GMP-compliant production of [^212^Pb]Pb-PSC-PEG_2_-TOC

**DOI:** 10.1186/s41181-024-00305-8

**Published:** 2024-11-27

**Authors:** Marc Pretze, Enrico Michler, David Kästner, Falk Kunkel, Edwin A. Sagastume, Michael K. Schultz, Jörg Kotzerke

**Affiliations:** 1 Department of Nuclear Medicine, University Hospital Carl Gustav Carus, Technical University Dresden, Fetscherstr. 74, 01307 Dresden, Germany; 2 Eckert & Ziegler Eurotope, 13125 Berlin, Germany; 3 Perspective Therapeutics, Coralville, IA 52241 USA; 4 Department of Radiology, The University of Iowa, Iowa City, IA 52240 USA; 5 University of Iowa, Iowa City, IA 52241 USA; 6 Department of Chemistry, The University of Iowa, Iowa City, IA 52242 USA

**Keywords:** ^212^Pb, TOC, Automation, GMP-compliant, Neuroendocrine tumour, Somatostatin receptor, Targeted alpha-therapy, VMT-α-GEN, VMT-α-NET

## Abstract

**Background:**

Recently, radiotheranostics comprising the true matched radionuclide pair ^203/212^Pb could serve as real dosimetric planning utility using ^203^Pb-radiolabelled pharmaceuticals before therapy with ^212^Pb-radiolabelled counterparts. ^212^Pb might act as the missing radionuclide therapy between standard β^–^ therapies (e.g. with ^177^Lu or ^90^Y), in some cases leading to β^–^ resistance and highly cytotoxic α therapies (e.g. with ^225^Ac) leading in some cases to renal insufficiency or even renal failure, due to the daughter nuclide ^213^Bi, which accumulates in > 90% within the kidneys during ^225^Ac therapy. ^212^Pb converts to ^212^Bi by β^–^-decay and the following pathway of decay bears in sum only one α decay, which certainly happens within the targeted tumour tissue. Following daughter nuclides (e.g. ^208^Tl), which could distribute in organs at risk have only β^−^ or γ decay, which is not as cytotoxic as α decay.

**Results:**

By ingenious customization of the standard cassettes of the ML EAZY it was possible to adapt the manual radiosynthesis of [^212^Pb]Pb-PSC-PEG_2_-TOC ([^212^Pb]Pb-VMT-α-NET) to a GMP-compliant synthesis module. The whole process of production, namely conditioning of C18 cartridge for purification, elution of the ^224^Ra/^212^Pb-generator, radiolabelling, C18 purification and sterile filtration performed automatically within one hour to access [^212^Pb]Pb-VMT-α-NET for patient application. [^212^Pb]Pb-VMT-α-NET was radiolabelled with high radiochemical purity > 95% and high radiochemical yield > 95% with molar activity ~ 15.8 MBq/nmol.

**Conclusions:**

The Lead-it-EAZY process performed stable and robust over ten radiosyntheses and yielded sterile [^212^Pb]Pb-VMT-α-NET in high purity for patient application. By changing the precursor this process could easily be adapted to other ^212^Pb-radiopharmaceuticals.

**Supplementary Information:**

The online version contains supplementary material available at 10.1186/s41181-024-00305-8.

## Background

**Fig. 1:**
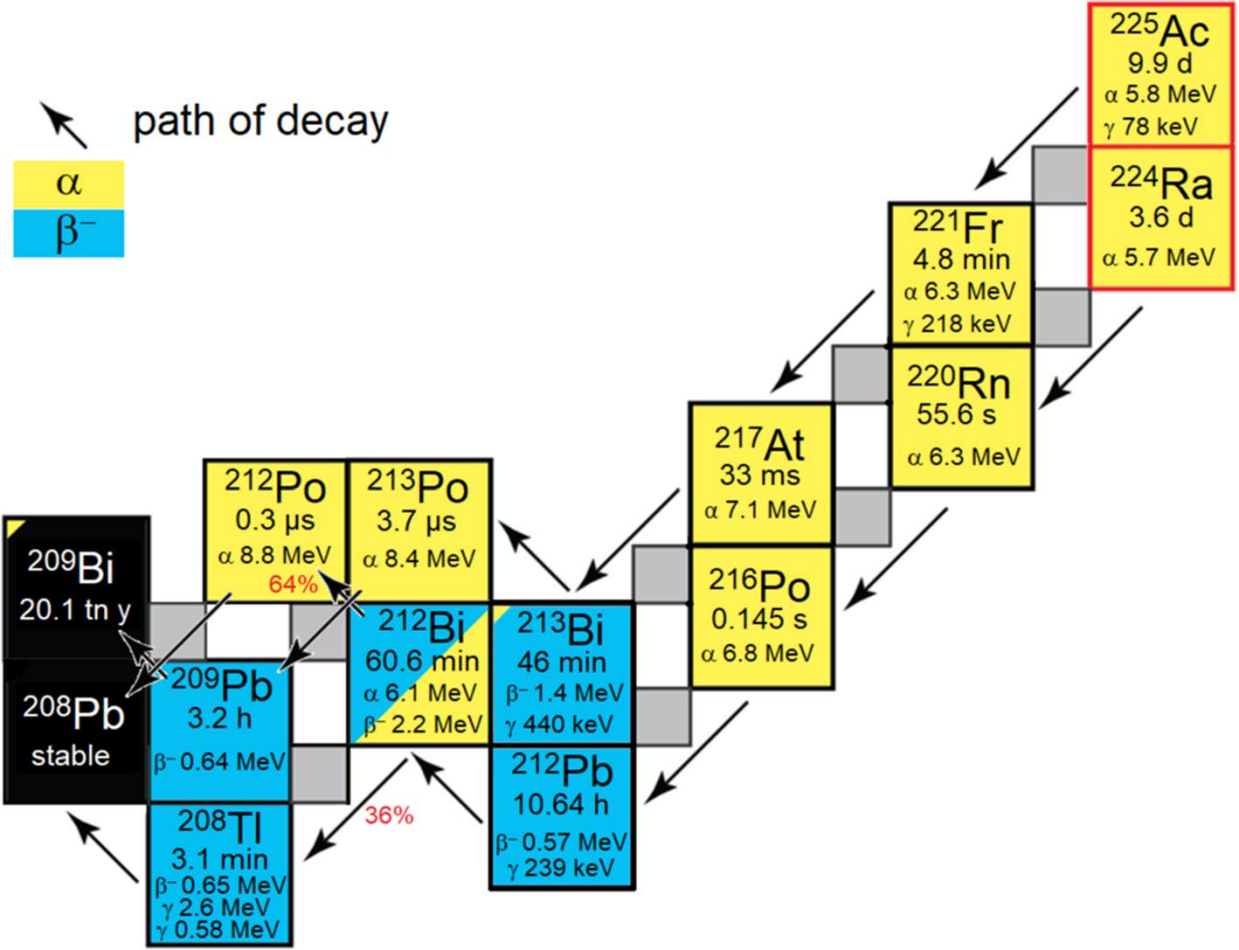
Decay diagram for ^225^Ac and ^224^Ra. Shown are their daughter nuclides with half-life (tn = trillion), type of decay and the corresponding decay energy and important γ energies for imaging and identification in γ spectrum analyses. The arrows indicate the main decay of the nuclide. ^212^Bi has two major decays, the probability of which is given in percent (red) by the arrows

Recently, receptor targeted α-therapy (TAT) has increased in importance in clinical routines of nuclear medicine, especially for tumour patients who develop resistance to β^–^ therapies (Miederer et al. 2024). Normally, patients receive multiple doses of ^90^Y or ^177^Lu (dose of 5–8 GBq per cycle) at periodic intervals of administration (e.g., 8-week intervals) (Brogsitter et al. 2017). Unfortunately, a substantial fraction of these patients experience progressive disease at some time and therapy is discontinued. On the other hand, it has been observed that further response and prolonged survival can be achieved by initiating α-therapy following disease progression. For example, the use of ^225^Ac (dose: 100 kBq/kg, four α-particles in the decay) can dramatically reduce the required level of administered radioactivity (by a factor of approximately 1000 compared to ^177^Lu). Figure 1 depicts the ^225^Ac decay chain with alpha energies in the range from 5.8 to 8.4 MeV (tissue ranges 47–85 μm) with a total deposited alpha energy of 27.6 MeV (Bruchertseifer et al. 2019). However, the behavior of α-particles in tumour cells is complicated by α-emitting radionuclide progeny in the ^225^Ac series. Of particular concern are ^221^Fr, ^217^At, ^213^Bi, and ^213^Po. A key issue is the biological fate of ^213^Bi (t_1/2_ 46 min), which is transported out of tumour cells (via escape itself or by escape of ^221^Fr or ^217^At) and mainly accumulates in the kidneys and delivers α-emitting ^213^Po dose to kidneys, which in turn could have a higher negative impact to the renal function (Kratochwil et al. 2021). Therefore, it is often suggested that only patients with an efficient renal function are reasonably considered eligible to receive ^225^Ac therapy, whereby patients with limited renal function may not be eligible to receive these therapies.

^212^Pb (t_1/2_ = 10.6 h) is a promising radionuclide for targeted alpha particle therapy, which decays with 100% from ^212^Pb to ^212^Bi by β^–^-decay (0.57 MeV) (Fig. 1). The pathway then splits either 64% to ^212^Po by β^–^-decay (2.2 MeV) and then 100% to ^208^Tl by α-decay (8.8 MeV) or 36% directly to ^208^Tl by α-decay (6.1 MeV) (Li et al. 2020; McNeil et al. 2023). Therefore, a ^212^Pb-labelled radiopharmaceutical, once accumulated in the tumour tissue, deposit its highest dose of one α-particle in sum specifically within the tumour cells, with lower probability of further α-decay occurring in healthy organs. Thus, ^212^Pb α-therapy represents a more favorable choice for cancer patients who are naïve to (or who have progressed on) β^–^-therapy, including patients with reduced renal function. Ongoing preclinical and clinical studies are investigating the potential of ^212^Pb-labelled peptides and antibodies (Santos et al. 2019) using activities in the range between the nuclides ^177^Lu and ^225^Ac.

Recently, the true matched pair ^203/212^Pb came into focus via several first in-human theranostic applications (Meredith et al. 2014; Delpassand et al. 2022; Müller et al. 2022). While ^203^Pb (t_1/2_ = 52 h; γ = 279 keV; 81% intensity) represents an ideal elementally-matched imaging surrogate (Nelson et al. 2023), ^212^Pb (γ = 239 keV; 44% intensity) itself can be used for post-treatment SPECT imaging (Mikalsen et al. 2023). Two first-in-human approaches with TOC and PSMA confirm this statement (Michler et al. 2024; Griffiths et al. 2024). A true matched pair could finally overcome differential pharmacokinetic/pharmacological properties observed between diagnostic and therapeutic radiotracers with unmatched pairs of radionuclides (Kotzerke et al. 2019).

The well-known chelator DOTA and its derivatives tend to lose up to 40% of ^212^Bi from its ^212^Pb-complexes due to β^–^-conversion accompanied by 38% conversion electrons and a cascade of Auger-Meitner electrons (Mirzadeh et al. 1993; Bauer et al. 2024). However, the chelator PSC forms highly stable complexes with ^203/212^Pb and stays > 90% intact even after β^–^-conversion to ^212^Bi if connected to the right PEG linker (Li et al. 2023). PSC-PEG_2_-TOC (VMT-α-NET) is a somatostatin receptor subtype 2 (SSTR2) targeting peptide of very high stability for the treatment of neuroendocrine tumours (NET) that shows rapid tumour accumulation, high tumour retention and fast renal excretion (Li et al. 2023). Furthermore, pretargeting concepts using [^212^Pb]Pb-DO3A-PEG_7_-Tz, to investigate murine xenografted PDAC model pretargeted with 5B1-TCO are also under development (Bauer et al. 2024).

Once the supply with ^212^Pb is secured by companies developing ^224^Ra/^212^Pb-generators (e.g. Perspective Therapeutics, AdvanCell), safe and reliable synthesis methods have to be developed to minimize waste production and protect environment and operator against contamination. Automated synthesis systems are ideal for GMP-compliant production in controlled and closed environments. Several automated systems for example from Elysia-Raytest (Derlin et al. 2020), IBA molecular (Decker and Turner 2012), iPHASE (Wichmann et al. 2021), Scintomics (Lindner et al. 2020; Acar et al. 2019), Trasis (Sørensen et al. 2020), and Eckert&Ziegler (Iori et al. 2017; Aslani et al. 2015), are currently available for diverse GMP-compliant diagnostic and therapeutic radiotracer production.

The aim of this work was to evaluate the efficiency and reliability of the radiosynthesis of ^212^Pb-labelled peptides and establish the translation of the synthesis to an automated synthesis platform (Modular-Lab EAZY, Eckert&Ziegler) for clinical routine production (Eryilmaz and Kilbas 2021). The ML EAZY is a very small cassette-based module operated with a GMP-compliant software (Pretze et al. 2016). After careful validation of the process [^212^Pb]Pb-VMT-α-NET is now safely available in reproducible radiochemical yield (RCY) and radiochemical purity (RCP) for tumour therapy in patients in accordance with the regulations of the German Pharmaceuticals Act §13.2b.

## Results

### Radiochemistry

Optimization of the manual radiolabelling is described elsewhere (Pretze et al. 2023). In this previous work, the investigated molar activity (A_m_) of 15–40 MBq/nmol showed the highest cell uptake. Lower precursor concentration increases the tumour cell uptake, but leads to significant lower RCY. For patient application, this A_m_ is maintained by using a precursor concentration of 0.1 µg/MBq of ^212^Pb for radiolabelling, resulting in stable RCY for different starting activities and a stable A_m_ of ~ 15.8 MBq/nmol.

The elution yield of the ^224^Ra/^212^Pb generator fluctuated between 59 and 80% (Table S1), depending on days between consecutive generator elution (Table 1). While automated procedure improves the radiation safety for operators, the elution efficiency is comparable to the manual elution (Fig. S1). 50 mg of Pb resin was adequate for trapping the activity of a fresh ^224^Ra/^212^Pb generator. Higher loading, e.g. 100 mg Pb resin needed more volume (3–4 mL) of Pb resin eluant and resulted in lower ^212^Pb and precursor concentration in the reaction vial, which led in consequence to lower RCY. For [^212^Pb]Pb-VMT-α-NET, HPLC is highly useful for determination of the identity of the product, but not for direct determination of RCY according to the approach used here. Only low amounts of free ^212^Bi together with higher amounts of free ^208^Tl daughter nuclides were observed in the TLCs to an extend of up to 20% 4 h after radiosynthesis. Importantly, the contribution of free ^208^Tl to the total body dose has been shown to be negligible (Orcutt et al. 2022).

**Table 1:** Manual (1^st^–6^th^) and automated (7^th^–27^th^) [^212^Pb]Pb-VMT-α-NET reactions

Day of Gen. arrival^1^	Eluted activity [MBq]	Elution yield [%]	Activity yield [MBq]	RCY [%]	RCP [%]^2^
1st	344*	62	247*	72	95
4th	297*	77	218*	73	91
5th	195*	61	183*	94	92
6th	164*	62	138*	84	93
7th	75	69	73*	97	94
12th	60	80	59*	98	93
14th	41	61	40*	98	97
15th	32	69	29*	91	92
18th	23	72	21*	91	92
19th	16	59	15*	94	94
20th	13	59	12*	92	95
25th	5.0	69	4.9*	98	94
27th	3.4	69	3.2*	94	93

^1^Reactions were always performed with 0.1 µg/MBq VTM-α-NET for 35 min at 105 °C. ^2^TLC with 0.1 M citrate pH 5. *Normalization was performed (see Table 2)

Measurements of the activity with a dose calibrator (ISOMED 2010, Nuvia Intruments GmbH, Dresden, Germany) show different values in eqilibrium of mother and daughter nuclides and after Pb-resin and C18 purification. Thus, a normalization factor for the dose calibrator is required immediately after each purification step (Table 2). The equilibrium of ^212^Pb with its daughter nuclides is reached 4 h after synthesis. The relatively short half-life of ^212^Pb leads to serious lower activity at the equilibrium (e.g. of 100 MBq at synthesis end are just 77 MBq left at equilibrium after 4 h). Therefore, the true activity of the freshly purified product must always be calculated using a normalization factor instead of waiting for equilibrium. However, the exact volumetric activity can already be measured by gamma spectrometry analysis of a defined volume (HPGe detector, e.g. 100 µL) of the product and integration of the ^212^Pb peak at 239 keV right after purification and formulation.

**Table 2:** Factor for normalization for activity measurement of ^212^Pb after separation from daughter nuclides via Pb-resin for the dose calibrator ISOMED 2010

Time after separation [min]	Normalization factor
0	1.86
5	1.80
30	1.58
60	1.37
90	1.26
120	1.18
180	1.10
240	1.06

Purification with C18 cartridges led to a further loss of product activity (6–10%). During manual syntheses the cartridge purifications were therefore found to be obsolete, due to optimized radiolabelling conditions. However, for the sake of GMP and automation, the C18 purification was implemented into the automated synthesis.

### Transfer of the manual process to modular-lab EAZY

For the adaption to the Modular-Lab EAZY, the following conditions were considered: The elution of the ^224^Ra/^212^Pb generator and concomitant trapping of the eluate onto Pb resin, as well as the moisturizing of the generator needs an additional 3-way valve module and a pump module, since the ML EAZY itself has to few ports for the whole generator elution, radiolabelling and purification process. The reaction time must be at least 35 min since the complexation of ^212^Pb needs longer time as compared to ^203^Pb, which only needs 15 min for complete complexation even at high activities > 2 GBq. This holds true for activity of > 500 MBq ^212^Pb and 50 µg precursor. If the amount of precursor drops < 16 µg (10 nmol) for activities < 100 MBq, the reaction kinetics might be influenced by the lower precursor concentration (Xia et al. 2020), but this was not observed here. Even though the optimal temperature for the reaction is 95 °C the setpoint for the reaction had to be increased to 105 °C to achieve optimal RCY with the automated method.

The Lead-it-EAZY-cassette with C18 purification consists of a modified standard cassette for labelling DOTA peptides with ^68^ Ga (Fig. S6). The reactor was replaced by a reactor from a C0-LUDOTAPEP-CM standard cassette. The ML EAZY has only one squeeze valve for two silicon tubes to transfer liquids either to waste vial or to product vial. Therefore, an innovative system of y-distributors and check valves was implemented in front of the squeeze valve in order to transport several different liquids either to waste vial or to product vial. All buffer and eluant vials are conic with a micropin hole (MP1000, B.Braun) in order to achieve quantitative liquid transfer. Buffer and eluants were added through these micropin holes by syringe and canula. For venting purposes, the canula should only situated halfway through the micropin holes. Further, the activation of the C18 cartridge is performed automatically within the whole process by rinsing the C18 with 1.5 mL 70% EtOH from the reactor, followed by rinsing the C18 with 3 mL 0.9% NaCl from the reactor, before elution of the ^224^Ra/^212^Pb generator. Residual EtOH and NaCl in the reactor do not disturb the subsequent radiolabelling. Sterican canula (4,665,791, B.Braun) may be used for transfer of liquids, as they are silicon-coated ensuring low metal input into the reaction solution. An ultra-low protein-binding sterile filter (vented SLGVV255F) was used for filtration, since the loss of product on this type of filter is < 3%. Complete transfer is achieved by this configuration as it tolerates intermediate gas flow through the vented filter. For a detailed reaction setting and outcome, see supporting information.

### Validation of the automated syntheses with C18 purification

**Fig. 2:**
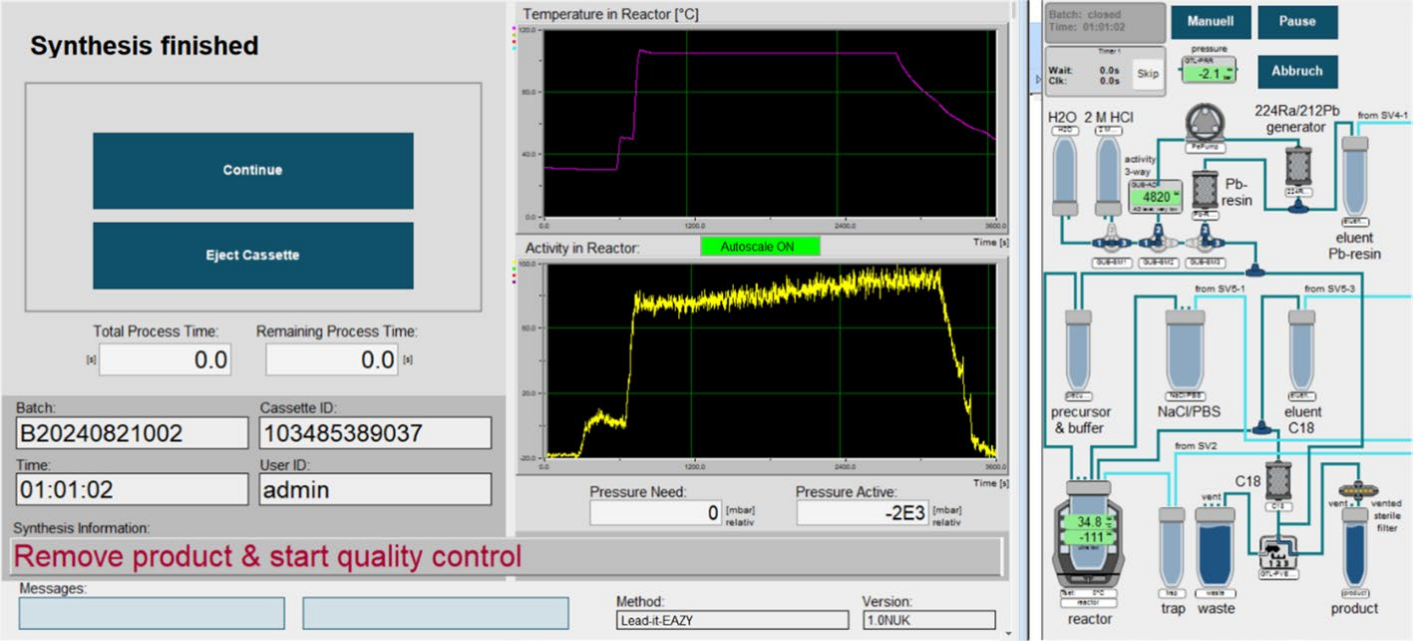
Schematic depiction and typical graphs for activity level and temperature of the Lead-it-EAZY process on the ML EAZY module

The automated method was transferred to the Modular-Lab EAZY and was tested for reproducibility, stability and transfer for routine production and the module was assembled as depicted in Fig. 2 and Fig. S7. After 10 syntheses, an optimal peptide concentration of 0.1 µg/MBq may be accepted. A complete reaction overview can be found in Table 1 and in the supporting information. The following acceptance criteria were used to decide for a successful automated production:

- RCP > 90% prospective (right after radiosynthesis), > 95% retrospective (if activity is < 90% prospective, a retrospective measurement 2 h later will be > 95% for ITLC-SG with 0.1 M citrate buffer pH 5, when the radiolabelling was successful)
- Endotoxin level < 5.00 EU/mL; < 175 EU for the whole product solution (Ph. Eur.)
- RCY 80–90%
- Product pH 4.0–8.0

Additionally, three validation batches for [^212^Pb]Pb-VMT-α-NET were performed without further change of liquid transfer times, to prove the reproducibility of the automated process.

## Discussion

Based on the data of 20 previously performed manual syntheses with four different ^224^Ra/^212^Pb generators, the following conclusions can be drawn:

The buffers can be created with reagents of the EZ-102 reagent kit. Namely, 1 M NaOAc (pH 4), 1 M NaOAc (pH 6) and 20% sodium ascorbate in H_2_O_Suprapur_ as well as 50% EtOH_abolute_. The Pb resin and the 2 M HCl_Suprapur_ have to be obtained from other sources. The buffer solutions can be stored for more than twelve months at 4 °C, enough time for the longevity of several ^224^Ra/^212^Pb generators.

Even with optimized process the following losses were observed: around 1–3% residual activity remained on the Pb resin, although it was rinsed with 2 mL buffer solution. Additionally, around 1–3% of activity remained in the reactor, although it was rinsed two times with saline. Further, around 3% activity remained on the purification cartridge and the filter. This leads to a loss of 7–9% of starting activity for the whole process. Therefore, an RCY of > 90% is reasonable.

The precursor concentration (0.1 µg/MBq) results in a maximum mass of 50 µg (31.7 nmol) for 500 MBq ^212^Pb-labelled peptide, which is half to the mass used for routine ^177^Lu-preparations with 5000 MBq (Brogsitter et al. 2017). At this concentration, any ^212^Bi generated during the reaction time by free ^212^Pb is rebound by the precursor (Li et al. 2023). Typically, doses of 1–1.5 MBq/kg were administered per patient. In individual patient application, this dose lead to a stable disease for more than six months, while only weak adverse effects were observed. The detailed results of patient treatments will be discussed in a further publication.

The necessity to use higher amounts of precursor as compared to diagnostic radiopharmaceuticals leads to complexation not only of ^212^Pb but also likely of its daughter nuclide ^212^Bi, which can be exploited for the quality control. If the quality control sample is taken quickly (< 1 min) from the final product and immediately submitted to TLC, the waiting time for the true RCP value was reduced to < 1 min, since the amount of free ^212^Bi and ^208^Tl is low (Fig. S2). If the quality probe is not taken immediately from the final solution (e.g. after 30 min), the newly generated free ^212^Bi (~ 50% of free ^212^Pb) and free ^208^Tl (~ 100% of bound ^212^Pb) prolonged the waiting time for true RCP values to > 60 min on TLC (Figs. S3 and S4).

Since the radiolysis and alpha decay is often discussed as fatal for the radiolabelled molecules in solution (Roscher et al. 2020), the stability of the ^212^Pb-labelled product was tested at room temperature. A TLC sample was taken from the product vial 4 h after radiosynthesis. Right after development of the TLC strip, > 20% of activity was found as unbound activity at the front of the TLC (Fig. S3). However, the activity of the spot for unbound radionuclides decreased over time and 10 h after development of the TLC strip, the spot for unbound radionuclides decreased to below 5% (Fig. S4), indicating no significant radiolysis of the ^212^Pb-labelled product within 4 h. The activity at the front of the TLC was mainly due to free ^208^Tl, which is reasonable due to 36% direct alpha decay from ^212^Bi.

## Conclusions

The Lead-it-EAZY process involves in brief the addition of eluant and buffers to the corresponding vials and the addition of EtOH to the reactor for automated C18 conditioning. The process might also be adapted to other precursors but the optimized concentration of precursor VMT-α-NET of 0.1 µg/MBq and the reaction time of 35 min at 105 °C should be validated before. Finally, the 60-min lasting process stably yielded [^212^Pb]Pb-VMT-α-NET with RCYs of 94.8 ± 3.0% and RCPs of 93.8 ± 1.6%. A reliable and safe method to produce ^212^Pb-peptides which can be carried out in closed compartments to avoid release of activity into the environment was described (e.g. ^220^Rn *Thoron*; will be discussed in another publication). The contaminated single-use cassette can be disposed after the synthesis. Considering routine production, a reduction of the dose for personnel to background radiation by factor four was achieved by the automated radiosynthesis.

## Materials and methods

All reagents and solvents were purchased in highest purity from commercial suppliers and were used without further purification. VMT-α-NET (VMT-α-NET) and ^224^Ra/^212^Pb-generator (VMT-α-GEN) were obtained from Perspective Therapeutics Inc. (Coralville, Iowa, USA). The Modular-Lab EAZY module (GTL) with software Modular-Lab v6.2 and reagent kits for cassette assembly were obtained from Eckert&Ziegler (Berlin, Germany). SepPak C18 light (WAT023501) cartridges were purchased from Waters (Milford, MA, USA). Custom-made Pb resin cartridges were filled with 50 mg powder (PB-B10-F, Triskem, Bruz, France). The dose calibrator (calibrated by a Cs-137 source AN-1426) and the CoMo-170 for separate α-detection and β/γ-detection were obtained from NUVIA Instruments. The HPGe detector GC2018 was purchased from Canberra (Rüsselsheim, Germany),

Solvents for quality control were stored at 4 °C. Buffer und precursor were stored at -20 °C; other chemicals were stored at room temperature. The pH was acquired by a QUANTOFIX Relax reflection photometer (91,346) with the corresponding pH test strips 5.5 × 85 mm pH-Fix 2.0–9.0 (92,118) (Macherey Nagel, Feucht, Germany). The endotoxin test device EndoSafe PTS was obtained from Charles River (Sulzfeld, Germany).

RCP was monitored by thin-layer chromatography (TLC) on iTLC-SG plates (Agilent, Santa Clara, California, USA). Measurement of the radionuclidic purity (RNP) and evaluation of the radio-TLC and was performed with a thin-layer scanner (MiniScanPRO + , Eckert&Ziegler Eurotope GmbH, Berlin, Germany) equipped with a Model 43–2 alpha detector ZnS(Ag) scintillator (Ludlum Measurements, Sweetwater, Texas, USA) and a built-in multi-channel analyzer for gamma spectroscopy.

Radio-HPLC was performed on a Shimadzu HPLC system (Shimadzu Deutschland GmbH, Duisburg, Germany), equipped with a reverse phase column (Analytical: Merck Chromolith HighResolution RP-18e; 150 × 4.6 mm plus a guard column 5 × 4.6 mm and a UV-diode array detector (220 nm). The solvent system used was a gradient of acetonitrile:water (containing 0.05% TFA) (0–8 min: 0–60% MeCN) at a flow rate of 2 mL/min unless otherwise stated.

### Radiochemistry

Automated radiolabelling of VMT-α-NET was performed according to established protocols for manual synthesis (Pretze et al. 2023). In brief, 0.1 µg/MBq of the precursor (M = 1578.7 g/mol) in H_2_O_Suprapur_ was added into a 5-mL-conic vial together with 100 µL EtOH_absolute_, 290 µL 1 M NaAc/AcOH buffer (pH 4, 99,99% trace metal) and 2 mg sodium ascorbate (Ph.Eur.) in 100 µl H_2_O_Suprapur_.

The automatic synthesis started with elution of the ^224^Ra/^212^Pb-generator with 4 mL 2 M HCl_Suprapur_ and ^212^Pb was trapped on a custom-made Pb-resin cartridge (50 mg PB-B10-F, Triskem, Bruz, France) preconditioned with 1 mL 2 M HCl_Suprapur_. The activity was eluted with 2 mL NaAc/AcOH buffer (pH 6, 99.99% trace metal) into the conic precursor/buffer-vial and from there directly into the reaction vial. The solution was heated at 105 °C for 35 min. Afterwards, the reaction was cooled for 5 min to 70 °C and the solution was diluted by addition of 2 mL 0.9% NaCl solution.

The cooled and diluted reaction solution was purified by slowly passing it over a C18 Plus light cartridge (WAT023501, Waters) automatically preconditioned with 1.5 mL 70% EtOH and 3 mL 0,9% NaCl. The C18 cartridge containing the product was rinsed with 2 mL 0.9% NaCl solution and was eluted with 1.5 mL 70% EtOH for injection through a vented sterile filter (SLGVV255F, Millex-GV, Merck) directly into a product vial. Finally, the product was diluted with 7 mL 0.9% NaCl solution to ensure the product with a pH ranging from 4.6 to 5.0 and to maintain an EtOH percentage below 10%.

The costs for the automated synthesis might be estimated as follows but are no guarantee: the cassettes would cost 200–250 € and the ML EAZY would cost ~ 30,000 €. To the date a price for the ^224^Ra/^212^Pb-generator and precursor VMT-α-NET is not established.

### Quality control of radiotracer

The quality control included several standard tests established in the clinical production:

- HPLC [^212^Pb]Pb-VMT-α-NET *t*_R_ = 5.4 ± 0.1 min (Fig. S1).
- TLC with eluant 0.1 M Na-citrate pH 5 (Start: [^212^Pb]Pb-VMT-α-NET and colloidal nuclides (R_*f*_ < 0,4), front: ^212^Pb-chloride) (Fig. S2).
- TLC with eluant 1 M NH_4_Ac: MeOH 1: 1 (Start: colloidal nuclides, front: [^212^Pb]Pb -VMT-α-NET and.^212^Pb-chloride) (Fig. S3).
- The pH was determined with the pH meter Quantofix: pH value: 4.9 ± 0.3.
- Radionuclide purity (RNP) and the exact volume activity were determined by a HPGe detector: ^212^Pb: 75 and 238 keV, ^212^Bi: 727 keV (6.7%), ^208^Tl: 510 (22.6%), 583 (85.0%) and 860 (12.5%) keV (Fig. S4).
- 10 µL of the product solution was diluted with 990 µL sterile water (1:100) and used for determination of the endotoxin level with EndoSafe PTS.

## Supplementary Information

Supplementary Material 1.

## Data Availability

All data is available on request from the corresponding authors.
