# Supplementary material for: Lead-it-EAZY! GMP-compliant production of [212Pb]Pb-PSC-PEG2-TOC
Source: EJNMMI Radiopharm Chem. 2024 Nov 27;9:81. doi: 10.1186/s41181-024-00305-8 (PMC11602913; doi:10.1186/s41181-024-00305-8)
Supplement: Supplementary file 1 — Supplementary Material 1. [file 41181_2024_305_MOESM1_ESM.docx]

Lead-it-EAZY! GMP-compliant production of [^212^Pb]Pb-PSC-PEG_2_-TOC

Marc Pretze ^1,#,^*, Enrico Michler ^1,#^, David Kästner ^1^, Falk Kunkel ^2^, Edwin E. Sagustume ^3^, Michael K. Schultz ^3,4,5,6^ and Jörg Kotzerke ^1^

^1^ Department of Nuclear Medicine, University Hospital Carl Gustav Carus, Technical University Dresden, Fetscherstr. 74, 01307 Dresden, Germany;

^2^ Eckert & Ziegler Eurotope, 13125 Berlin, Germany; Falk.Kunkel@ezag.de

^3^ Perspective Therapeutics, Coralville, Iowa 52241, USA;

^4^ Department of Radiology, The University of Iowa, Iowa City, IA 52240, USA;

^5^ Interdisciplinary Graduate Program in Human Toxicology, University of Iowa, Iowa City, Iowa 52241, USA;

^6^ Department of Chemistry, The University of Iowa, Iowa City, Iowa City, IA 52242, USA;

^*^ Correspondence: marc.pretze@ukdd.de; Tel.: +49 351 458 5417

^#^ These authors contributed equally

**Supporting information**

**Step-by-step description of the automated ^212^Pb-peptide synthesis**

A ^224^Ra/^212^Pb generator with a starting activity of 550 MBq ^212^Pb in the first elution was obtained from Perspective Therapeutics (Coralville, Iowa, USA).

- Precondition Pb resin with 1 mL 2 M HCl_Suprapur_ (wet condition) and attach on the top right 3-way-valve.
- Add 4 mL 2 M HCl_Suprapur_ and 6 mL air to a 10-ml-syringe and attach on top of the left 3-way-valve.
- Add 1 mL H_2_O_Suprapur_ and 2 mL air to a 3-ml-syringe and attach on the left port of the left 3-way-valve.
- Connect C18 cartridge to the cassette.
- Add 1.5 mL 70% EtOH_absolute_ to vented reactor for automated C18 conditioning.
- Add 1.5 mL 70% EtOH_absolute_ to red-capped vial.
- Mix peptide with buffer, sodium ascorbate and EtOH_absolute_ in syringe and add solution to gold-capped vial
- Attach a vented sterile filter to a vented product vial and connect with product line.
- Attach a fresh 20-ml-NaCl (0.9%) or PBS buffer vial to the cassette.
- Start Software

1^st^ minute: the C18 is preconditioned with 1.5 mL 70% EtOH for 80 seconds.

3^rd^ minute: the C18 is preconditioned with 3 mL 0.9% NaCl for 40 seconds.

4^th^ minute: the ^224^Ra/^212^Pb generator is eluted with 4 mL 2 M HCl onto Pb resin for 320 seconds.

9^th^ minute: the ^224^Ra/^212^Pb generator is moistened with 1 mL H_2_O_Suprapur_ for 110 seconds.

11^th^ minute: the reactor is set to preheat the reactor to 50°C and the ^212^Pb is eluted with 2 mL 1 M NaOAc from Pb resin through the buffer-precursor mixture vial and further to the reaction vial for 110 seconds.

13^th^ minute: the reaction at 105°C for 35 minutes.

47^th^ minute: the reactor is switched off for 5 minutes to cool.

52^nd^ minute: the reactor has cooled to 70°C and 4 mL of saline is added to the reactor and the diluted reaction solution is transferred through the C18 cartridge to waste for 140 seconds.

56^th^ minute: 2 mL of saline is added to the reactor to rinse residual activity and the solution is transferred through the cartridge for 40 seconds.

57^th^ minute: the product is eluted from the C18 cartridge with 1.5 mL eluant through vented sterile filter to the product vial for 100 seconds.

59^th^ minute: 7.5 mL saline are added to the reactor for dilution of the product for 40 seconds.

60^th^ minute: the saline from the reactor is completely transferred through the C18 and the vented sterile filter to the product vial for 50 seconds.

61^st^ minute: the product is ready for quality control. A normalization factor of 1.8 has to be used for determination of the true activity.

The content of EtOH in the final injection solution is 9%. After 61 min synthesis time the RCYs are 94.8±3.0% with RCPs >93.8±1.6%. During manual synthesis, the dose for the personnel was 4–8 µSv in 1 h, which is four times of the normal background radiation. In automated synthesis, the dose for the personnel was 1–2 µSv in 1 h, which is the normal background radiation.


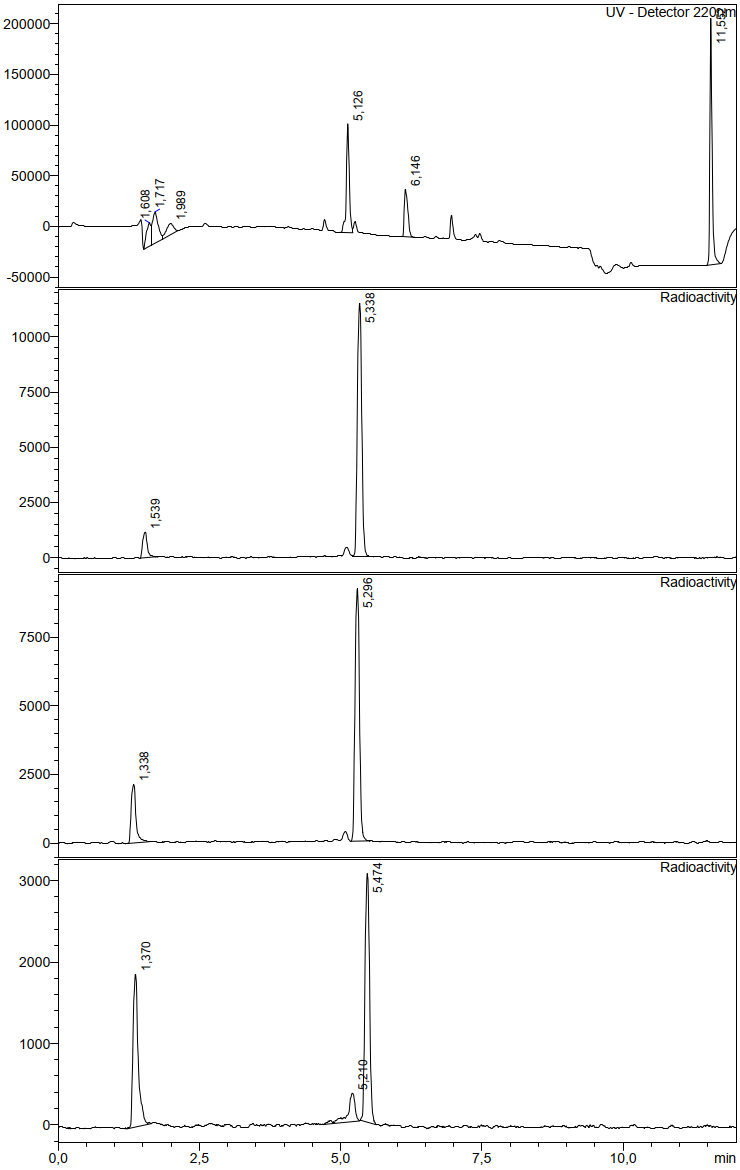


A

B

D

C

**Figure S1.** Representative analytical (radio-)chromatograms with variation of radiochemical purity of the ^212^Pb-conjugate over time: after 5 min **A)** UV trace (220 nm) after 5 min: precursor PSC-PEG_2_-TOC at *t*_R_ = 5.1 min, degraded precursor fragments at *t*_R_ = 6.1 and 7.1 min; **B)** gamma trace after 5 min: free ^208^Tl at *t*_R_ = 1.4 ± 0.1 min, [^212^Bi]Bi-PSC-TOC at 5.2 ± 0.1 min, 90.6% [^212^Pb]Pb-VMT-α-NET at *t*_R_ = 5.4 ± 0.1 min; **C)** gamma trace after 2 h: 79.8% [^212^Pb]Pb-VMT-α-NET; **D)** gamma trace after 16 h: 52.5% [^212^Pb]Pb-VMT-α-NET.


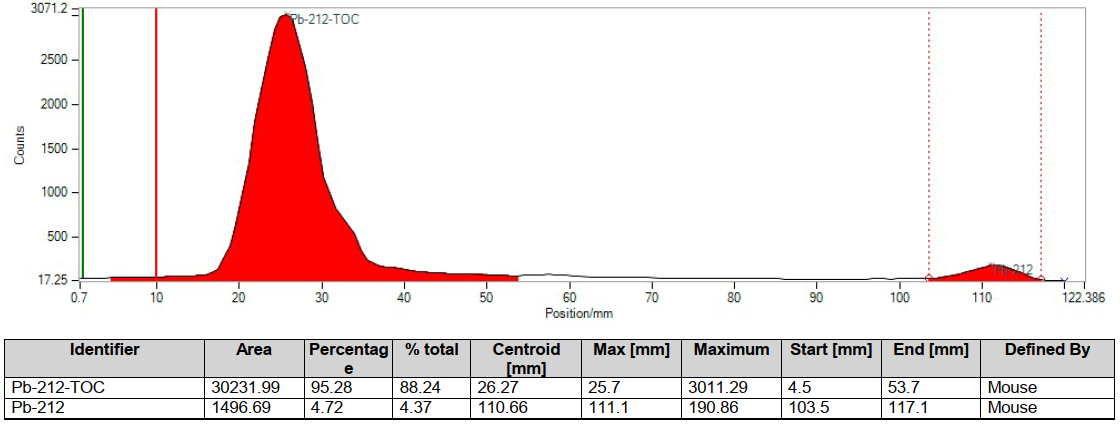


**Figure S2.** Representative analytical thin-layer chromatogram 5 min after synthesis – measured directly after TLC development with citrate eluant


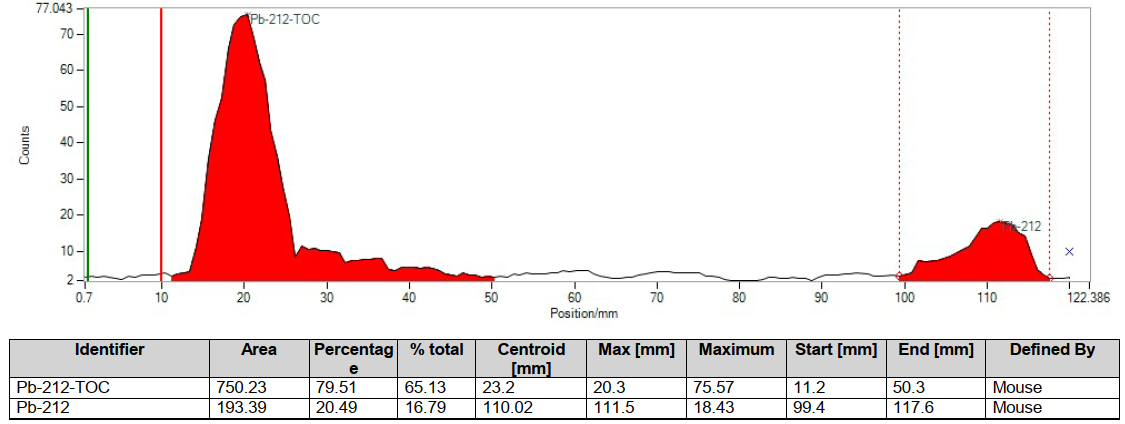


**Figure S3.** Representative analytical thin-layer chromatogram: probe sampling 4 h after synthesis indicating free ^212^Bi and ^208^Tl at front – measured directly after TLC development with citrate eluant


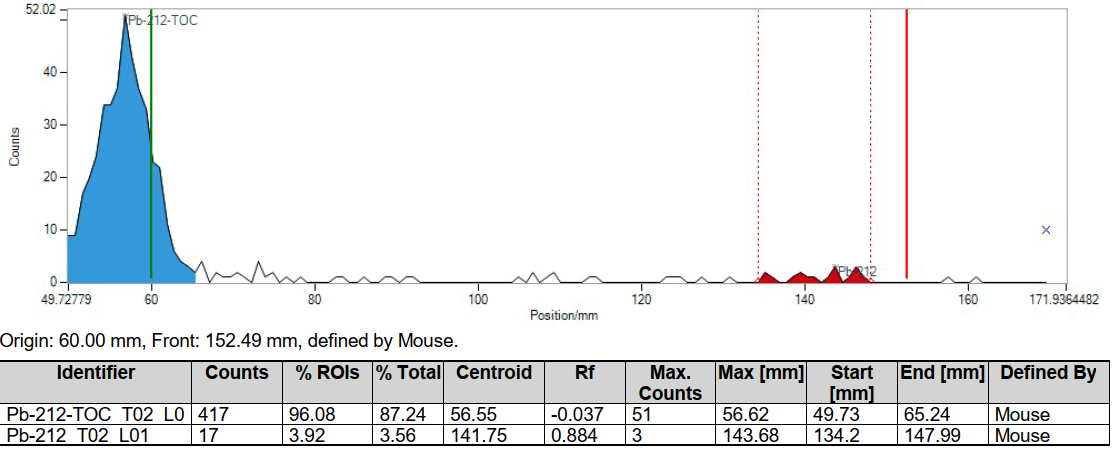


**Figure S4.** Representative analytical thin-layer chromatogram: probe sampling 4 h after synthesis – measured 10 h after TLC development with citrate eluant – free ^208^Tl and ^212^Bi at front are decayed


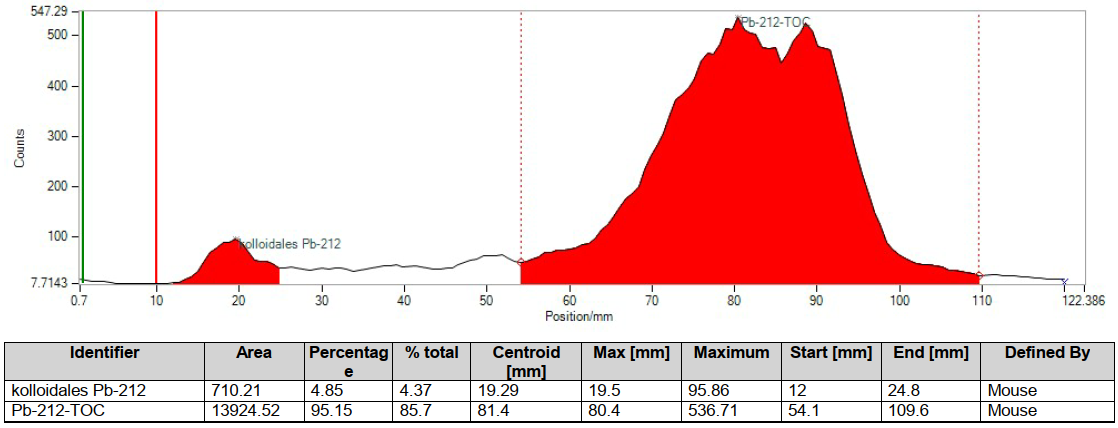


**Figure S5.** Representative analytical thin-layer chromatogram for detection of colloidal radionuclides 5 min after synthesis – measured directly after TLC development with 1 M ammonium acetate:methanol (v:v = 1:1) eluant


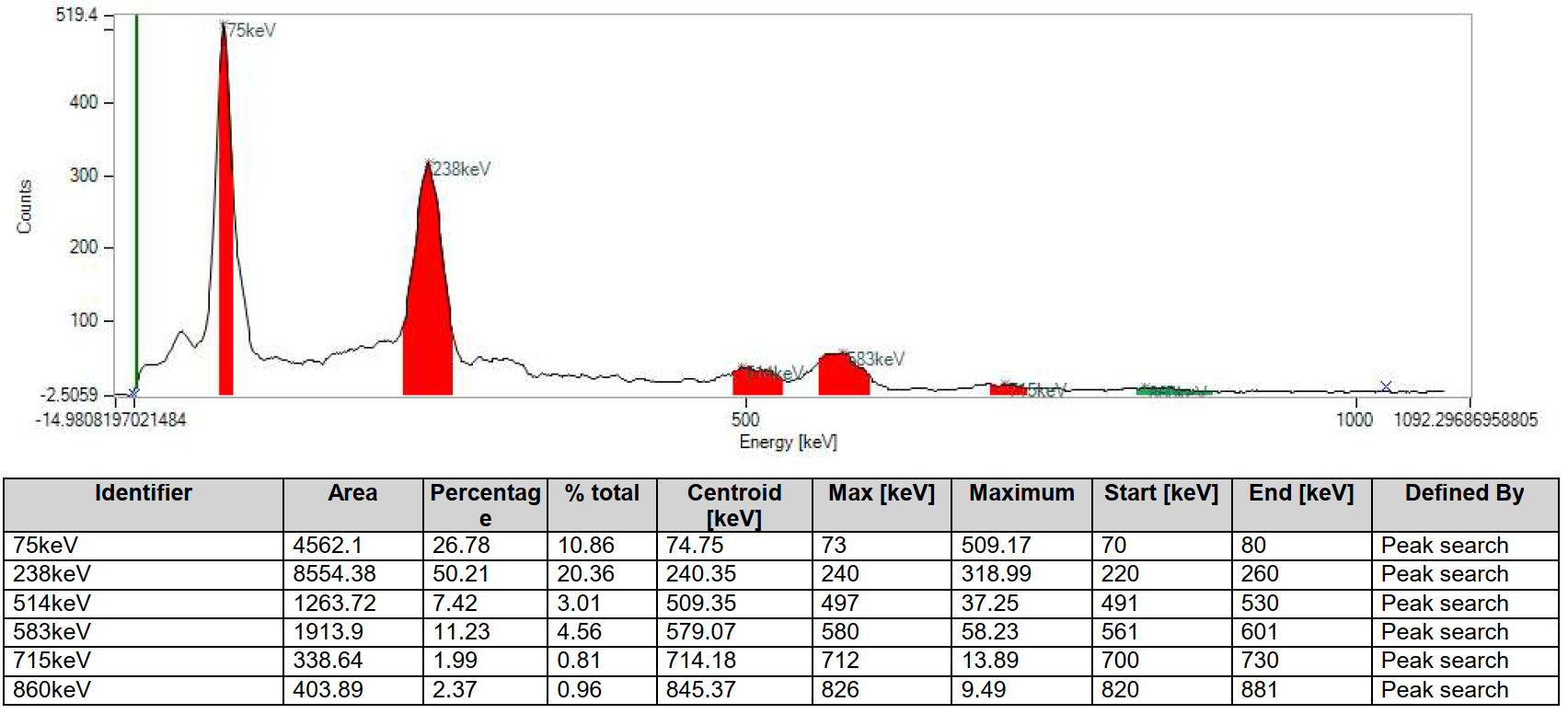


**Figure S6.** Respective gamma spectrum via MCA for verification of the RNP. ^212^Pb: 75 and 238 keV, ^212^Bi: 727 keV (6.7%), ^208^Tl: 510 (22.6%), 583 (85.0%) and 860 (12.5%) keV.


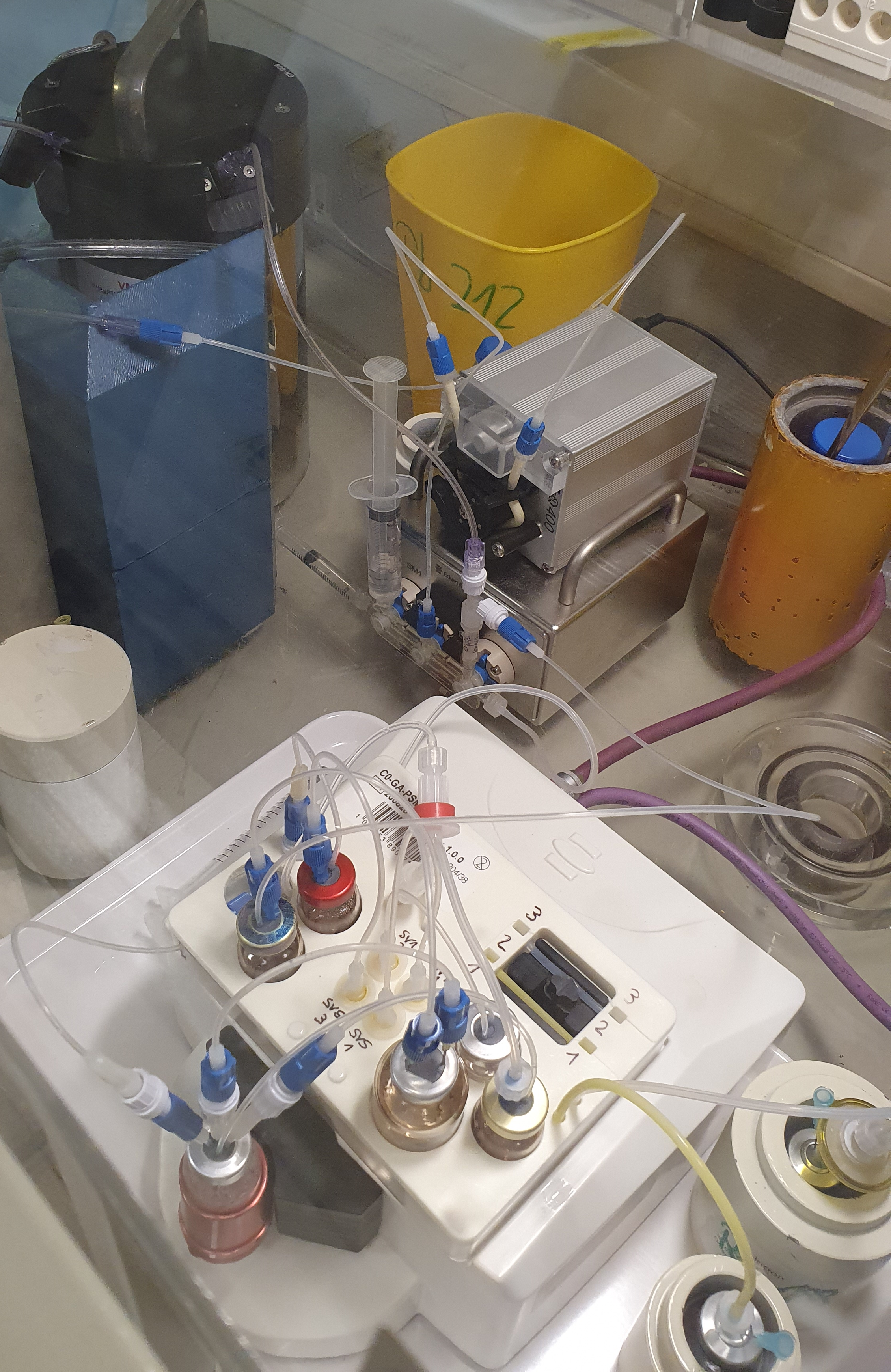


**Figure S7.** The Modular-Lab EAZY module in its final configuration including C18 purification. At the upper left is the ^224^Ra/^212^Pb-generator VMT-α-GEN. In the middle are the 3-way-valve and pump modules with HCl (big) and H_2_O (small) syringes for automatic generator elution. At the left side of the cassette are the Pb resin eluant vial (**blue cap**) and the C18 eluant vial (**red cap**). At the bottom in the middle of the cassette is the reactor (**dark red mantle**). At the bottom right of the cassette are the 20-ml-saline vial (**silver cap**) and the buffer/peptide vial (**gold cap**). At the bottom on the right is the waste vial, and on the right side with filter is the product vial.

**Table S1.** Elution efficiency in percentage of total activity obtained via manual elution of VMT-α-GEN ^224^Ra/^212^Pb radionuclide generator.

| Elution No.\Batch | VMT072IN23A | VMT226IN23B | VMT331IN23B | VMT205IN24A* |
| --- | --- | --- | --- | --- |
| 1 | 79% | 77% | 69% | 62% |
| 2 | 86% | 56% | 78% | 77% |
| 3 | 81% | 82% | 54% | 61% |
| 4 | 65% | 64% | 63% | 62% |
| 5 | 64% | 64% | 77% | 69% |
| 6 | 86% | 61% | 86% | 80% |
| 7 | 78% | 83% | 61% | 71% |
| 8 | 73% | 69% | N/A | 61% |
| 9 | 84% | 82% | N/A | 69% |
| 10 | 62% | 91% | N/A | 78% |
| Mean | 76% | 73% | 70% | 69% |
| Standard Deviation | 9% | 12% | 11% | 7% |

*As calculation example VMT205IN24A eluted 1202 MBq on the day of production. This value was taken as 100% ^224^Ra/^212^Pb. On arrival three days later there should be 681 MBq ^224^Ra left on the generator: 424 MBq (62%) ^212^Pb were eluted; after three further days of regeneration 385 MBq ^224^Ra should be left on the generator: 297 MBq (77%) ^212^Pb were eluted; after 1 further day of regeneration 319 MBq ^224^Ra should be left on the generator: 297 MBq (61%) ^212^Pb were eluted.
